# Test and Numerical Simulation Investigation on Seismic Performance of Different Types of Expansive Polystyrene Granule Cement Latticed Concrete Walls

**DOI:** 10.3390/ma14113082

**Published:** 2021-06-04

**Authors:** Xinyu Cao, Xiaojun Li, Baizan Tang

**Affiliations:** 1 Faculty of Architecture, Civil and Transportation Engineering, Beijing University of Technology, Beijing 100124, China; xinyu2016@emails.bjut.edu.cn; 2 Institute of Geophysics, China Earthquake Administration, Beijing 100081, China; 3 Engineering Research Center of Railway Environmental Vibration and Noise, Ministry of Education, East China Jiaotong University, Nanchang 330013, China; tangbaizan@ecjtu.edu.cn

**Keywords:** diagonal bracing, latticed concrete wall, expansive polystyrene granule cement (EPSC), quasi-static test, seismic performance, numerical simulation

## Abstract

An expansive polystyrene granule cement (EPSC) latticed concrete wall with diagonal bracing is formed with a traditional EPSC latticed concrete wall skeleton with added diagonal bracing. It is a new model of non-demolding wall integrating insulation and structure. For the new model, the length of one EPSC panel is 1200 mm, which is 300 mm longer than that of the traditional one. The diagonal bracing is arranged in a 45° orthogonal grid in the new model. In contrast, the traditional type has only horizontal lattice beams and vertical lattice columns. Through the pseudo-static test of two new EPSC latticed concrete wall specimens with diagonal bracing and two traditional EPSC latticed concrete wall specimens, the seismic performance of latticed concrete walls was investigated in this study. The main difference between the specimens was the lattice form and the core hole diameter. Finite element simulation was carried out on the simplified models of a latticed concrete wall with diagonal bracing. The results showed that EPSC could work with post-poured concrete to withstand earthquake action together. Additionally, the lateral performance of the EPSC latticed concrete wall with diagonal bracing was significantly improved compared with the traditional type, and the overall seismic performance was improved, especially the energy dissipation capacity, which increased by more than 180%. The bearing capacity increased by more than 12%, when the amount of concrete was basically the same. The initial stiffness was improved by more than 52%. As the diameter of the core hole increased 20 mm, the bearing capacity improved more than 12%. Simplified modeling methods could be used to analyze the seismic performance of latticed concrete walls under lateral cyclic loading. The study reveals the seismic performance characteristics of latticed composite walls with different lattice forms and core hole diameters, and it provides technical support for the engineering application of different lattice forms and core hole diameter latticed composite walls.

## 1. Introduction

The energy consumption of the construction industry has increased, and environmental pollution is occurring frequently [1,2,3,4,5,6]. Green low-carbon buildings have become an important trend [7,8,9,10,11]. Non-demolding wall integrated building insulation and structure is an important way to develop green buildings [12,13]. Traditional EPSC latticed concrete walls are non-demolding composite walls with integrated insulation and structure, consisting of factory-produced EPSC, steel bars, and post-pouring concrete [14]. EPSC has a variety of specifications in order to meet various actual engineering needs. EPSC latticed concrete walls are mainly used in the structures of villages and towns, and these walls can improve seismic performances and promote building energy savings [15,16]. When oblique bracing is added to a traditional EPSC latticed concrete wall, the wall is an EPSC latticed concrete wall with diagonal bracing.

The testing and numerical simulation research results have been obtained for the seismic performance of a latticed wall. Fu et al. [17] conducted a quasi-static test on a composite shear wall composed of a reinforced concrete (RC) grid frame and load-bearing blocks, and they found that the wall had good ductility and energy dissipation capacity. Lu et al. [18] carried out a pseudo-static test on a wall with a new type of grid frame structure composed of a reinforced concrete grid frame and cast-in-situ phosphogypsum, and they found that the structure had good energy dissipation capacity. Ma and Jiang [18] studied the seismic behavior of three new gypsum-concrete composite exterior wallboards with a quasi-static test. Based on the test [19], Jiang and Liang [20] studied the restoring force model of a new gypsum-concrete composite exterior wallboard. An expandable polystyrene (EPS) insulation block produced in the factory was bricked into a wall, with steel bars placed and cast-in-place concrete made up of a concrete grid wall with insulation. Through a series of studies [21,22], it was found that setting composite columns and increasing the axial compression ratio could improve the bearing capacity of the wall. Zhang et al. [23] adjusted the grid column center spacing of the composite wall to two times that of the original, in order to apply the concrete grid wall to rural buildings. Zhao [24] optimized the design of thermal insulation masonry in order to improve the seismic performance and energy saving ability of an RC grid frame with an insulating form. Han et al. [25] carried out the quasi-static test of precast concrete hollow shear wall specimens, and they found that the elastic-plastic deformation could meet the requirements of rare earthquakes. Dusicka and Kay [26] conducted pseudo-static tests on an insulated concrete form grid (ICFG) wall to study the influence of the aspect ratio and the axial compression ratio on the seismic performance of the wall. Based on the existing pseudo-static tests, Asadi et al. [27] conducted a finite element simulation analysis on the seismic performance of a screen-grid insulating concrete form (SGICF) wall. Further considering the sustainable development of building materials and the recycling of construction waste, Cao’s research team [28,29,30] conducted a serial pseudo-static test to analyze recycled concrete shear walls with thermal insulation blocks of single-row steel reinforcement and a specially shaped frame suitable for rural buildings, and they found that the system had good seismic performance. Cao et al. [31] conducted cyclic lateral loading on steel tubular rein-forced latticed concrete walls with different aspect ratios to explore the seismic performance and optimal design of the walls. Tang et al. [32,33,34] investigated the seismic performance of RC frame structures with full-scale EPSC latticed concrete infill walls, and they determined that these structures had good seismic performance through shaking table tests and numerical simulations. In the existing investigations, the lattice beams of the latticed concrete walls are mostly horizontal, and the lattice columns are mostly vertical, and the diameter of the core hole is relatively simple. It is necessary to carry out investigations into the seismic performance of latticed concrete walls with different lattice types and different core hole diameters.

In this research, quasi-static tests and numerical simulation methods were used to investigate the seismic performance of EPSC latticed concrete walls with different lattice types and different core hole diameters. Low cyclic lateral loading was applied to four EPSC latticed concrete walls with different lattice types and core hole diameters to evaluate the seismic performance of the walls through comparing and analyzing the damage characteristics, hysteresis performance, bearing capacity, ductility, stiffness degradation, and energy dissipation capacity. A reasonable simplified simulation method was obtained through numerical simulation analysis. The investigations were expected to provide a reference for the design and application of lattice concrete walls.

## 2. Materials and Methods

### 2.1. Design and Materials of Test Specimens

**Figure 1:**
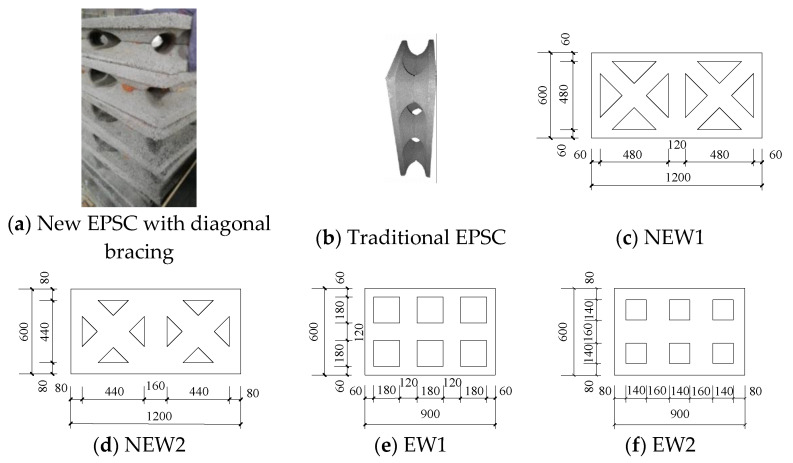
Schematic diagram of EPSC.

**Figure 2:**
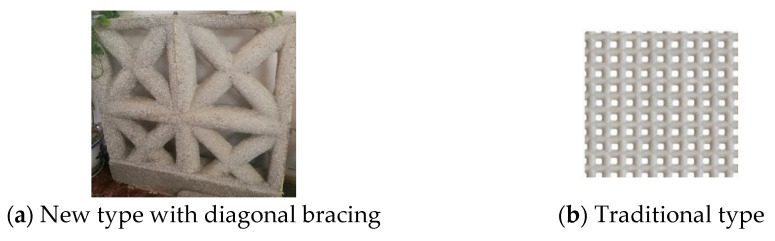
The skeleton of the latticed concrete wall.

Four full-scale EPSC latticed concrete wall specimens were designed as NEW1, NEW2, EW1, and EW2. For the specimens, the differences were considered, including the lattice types and core hole diameters. The core hole diameter of specimens NEW1 and EW1 was 120 mm, and the core hole diameter of specimens NEW2 and EW2 was 160 mm. For the new EPSC with diagonal bracing, whose core hole diameter was 120 (160) mm, the dimensions of the EPSC were 1200 mm (length) × 600 mm (height) × 210 (250) mm (thickness). For the traditional EPSC, whose core hole diameter was 120 (160) mm, the dimensions of the EPSC were 900 mm (length) × 600 mm (height) × 210 (250) mm (thickness). As shown in Figure 1, for the new and traditional latticed concrete walls, the spacings between lattice beams or lattice columns were 600 mm and 300 mm, respectively. The EPSC material properties are shown in Table 1. Figure 2 shows the skeletons of two types of latticed concrete walls.

**Figure 3:**
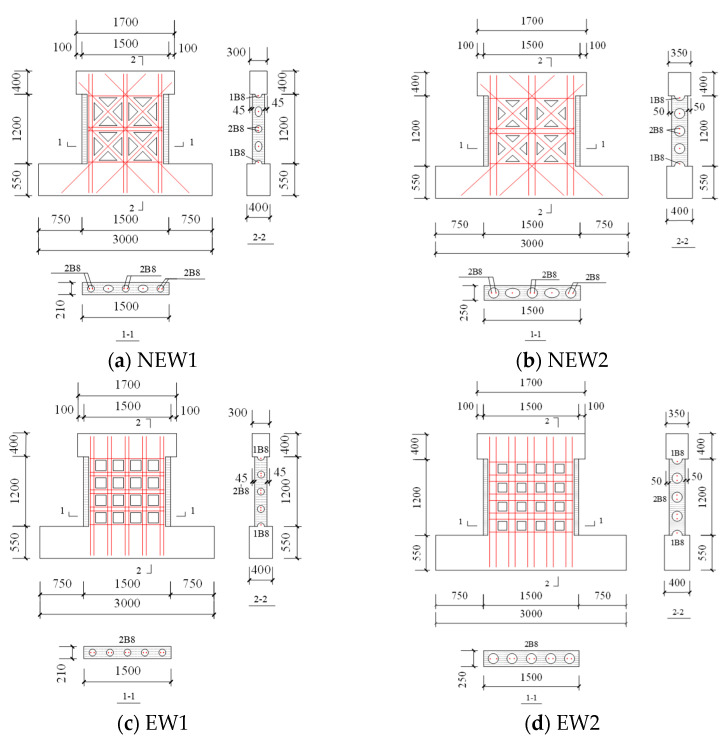
Wall specimen details (mm).

The reinforcement with a tensile strength design value of 300 N/mm^2^ was adopted and labelled as B. The concrete lattice beam (column) was reinforced with two steel bars with a diameter of 8 mm, and the concrete diagonal bracing was reinforced with one steel bar with a diameter of 8 mm. Figure 3 summarizes the dimensions and reinforcements of the specimens. Considering the comparative analysis of the test results, the amount of concrete and steel bars per unit area of the specimen with the same core hole diameter was essentially the same for different lattice types. The cubic compressive strength of the concrete was 20.8 MPa [35], as Grade C20 concrete was used for building the EPSC latticed concrete walls in the structures. The main parameters are shown in Table 2.

### 2.2. Test Setup

**Figure 4:**
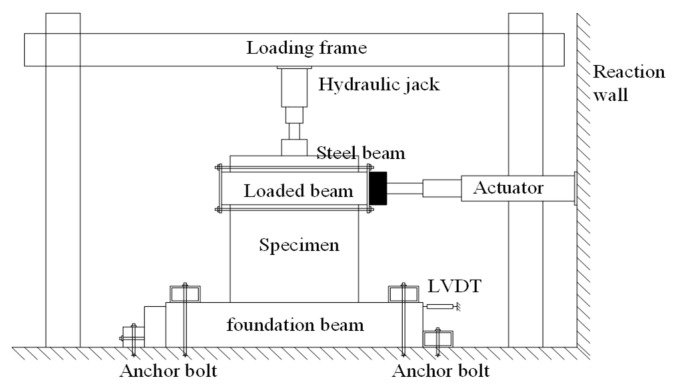
Loading device of the test.

The pseudo-static test method was adopted for the test. The test setup is illustrated in Figure 4. The vertical loading was applied by a hydraulic jack. During the test, the vertical load was applied to the distribution beam on the upper part of the loading beam of the specimen so that the axial compression ratio is 0.1, and this ratio remained unchanged during the test in order to simulate the load on the upper part of the wall. The horizontal loading was applied by the horizontal jack. During the test, the horizontal jack acted on one end of the loading beam to simulate the earthquake action. The foundation beam of the specimen was fixed by four high-strength ground anchor bolts to prevent the specimen from slipping during the test.

### 2.3. Test Program and Instrumentation

**Figure 5:**
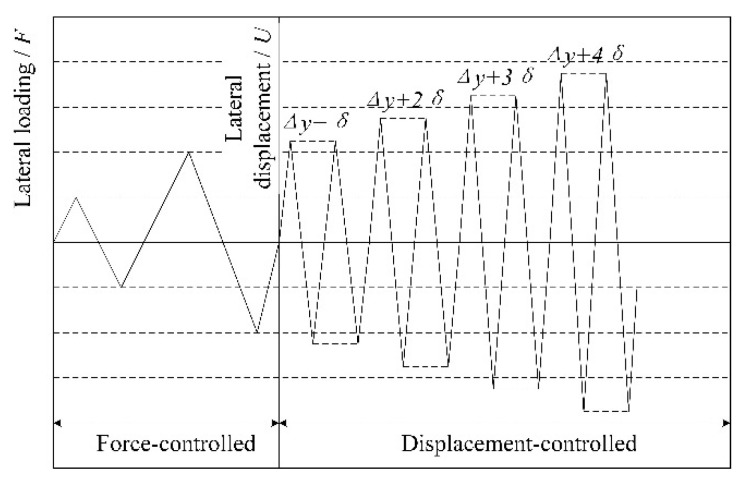
Low-reversed cyclic loading law.

For specimen EW1, the axial force was 120 kN, while the axial force of specimen EW2 was 210 kN. For specimen NEW1, the axial force was 100 kN, while the axial force of specimen NEW2 was 170 kN. The pseudo-static test adopted the force-displacement mixed control loading system [36]. Cyclic lateral force was applied step by step before the specimen yielded, with 10 kN adopted as the interval. The displacement controlled stage adopted multiples of the yield displacement as the interval after the specimen yielded. For specimens EW2 and NEW2 with 160 mm core hole diameter, 1 mm for early stage and 1.5 mm for later stage of displacement control interval were adopted respectively. For specimens EW1 and NEW1 with 120 mm core hole diameter, 1 mm was used as the interval. This was repeated twice at each step during the displacement control stage until the lateral force of the specimen dropped below 85% of its peak force or the specimen could not continue to be loaded safely. Low-reversed cyclic loading law is shown in Figure 5.

**Figure 6:**
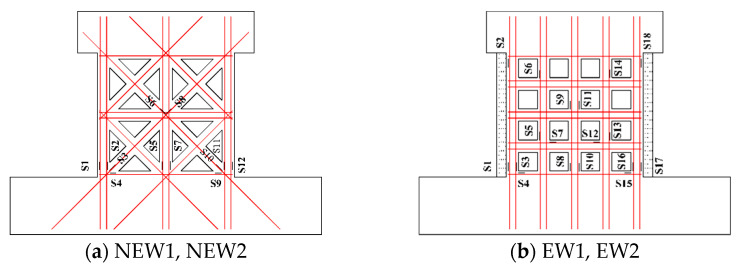
Arrangement of strain gauges of specimens.

A linear variable displacement transducer (LVDT) was arranged on the top surface of the foundation beam to measure the slippage. The steel strain gauge measured the strain of the steel during the test and it was numbered S, as illustrated in Figure 6. The layouts of the steel strain gauges of the new and traditional type specimens were the same.

## 3. Results and Discussion

### 3.1. Failure Mode and Crack Distribution

**Figure 7:**
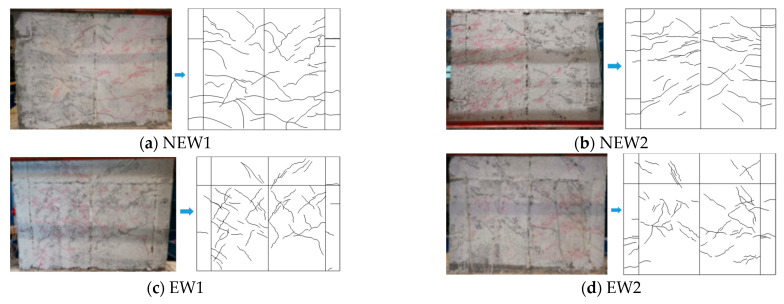
Failure modes of the specimens.

Specimen NEW1 was a new specimen type whose core hole diameter was 120 mm with diagonal bracing, which exhibited a shear failure mode. When pushed to 50 kN, a crack first appeared on the surface of the EPSC, and this crack was a horizontal crack at the corner of the wall. With the increase in the lateral load, the horizontal and oblique cracks in the middle and lower parts of the wall were gradually generated and extended, and the crack lengths were generally in the range of 100–300 mm. When pushed to 70 kN to draw the cracks, the sound of “rustling” of the wall could be heard. After this, the cracks in the middle of the wall were extended and developed. Displacement control was adopted after the specimen yielded. When the top lateral displacement was pushed to 9 mm (*θ* = 0.643%), an “X”-shaped shear crack appeared. The number of diagonal cracks increased and extended to the upper part of the wall. After that, “X”-shaped cracks were formed in the upper part of the wall, and the cracks gradually widened. When the top lateral displacement reached 14.91 mm (*θ* = 1.065%), the horizontal and diagonal cracks fully developed with the maximum width approximately 5 mm, the small pieces of EPSC at the bottom of the lattice side column were crushed and peeled off, and the test stopped. The final failure mode of the specimen NEW1 is shown in Figure 7a.

The new specimen type NEW2 had a core hole diameter of 160 mm with diagonal bracing, which was characterized by shear failure. When pushed to 40 kN, the first horizontal crack appeared in the lower part of the specimen, which was delayed compared with specimen NEW1. With the increase in the lateral load, similar to the crack development rule of specimen NEW1, the diagonal cracks in the lower part of specimen NEW2 increased and extended to the middle of the wall, and the cracks had a tendency to extend from the lower part of the specimen to the upper part. When pulled to 110 kN, diagonal cracks appeared on the upper part of the specimen. Subsequently, the loading system was changed to displacement control. When the top lateral displacement *δ* reached 11.5 mm (*θ* = 0.812%), multiple “X”-shaped cracks were approximately formed on the wall, indicating that the stiffness of the specimen was significantly degraded. At that time, the displacement angle was greater than that of specimen NEW1, which indicated that the increase in the core hole diameter could delay the damage of the specimen. When pushed, the top lateral displacement reached 16.02 mm (*θ* = 1.144%), the cracks had been fully extended, the width had increased, and the “X”-shaped cracks on the wall had clearly developed. The width of the horizontal crack at the root of the wall reached about 6 mm, and a small piece of the EPSC was crushed and peeled off. Then the loading stopped. The final failure mode and the crack distribution of the NEW2 specimen are shown in Figure 7b.

The EW1 traditional specimen had a core hole diameter of 120 mm with shear failure characteristics. When the lateral load was pushed to 30 kN, horizontal cracks first appeared in the lower part of the wall, and this first crack appearance was earlier than that of the new specimen NEW1 with diagonal bracing (50 kN). When loaded to 50 kN to draw wall cracks, the sound of “rustling” on the wall could be heard. However, for the NEW1 specimen, the load for this phenomenon was 70 kN. These two phenomena both indicated that the seismic performance of the new specimen with diagonal bracing was improved compared with the traditional specimen. During the displacement control, long diagonal cracks appeared. The crack width gradually increased. When the top lateral displacement *δ* reached 9 mm (*θ* = 0.643%), an “X”-shaped crack had formed on the wall. When the top lateral displacement *δ* reached 11.17 mm (*θ* = 0.798%), a small piece of EPSC at the root was crushed by extrusion and the maximum crack width reached approximately 5 mm. The final failure mode of specimen EW1 is shown in Figure 7c.

The EW2 traditional specimen had a core hole diameter of 160 mm with a shear failure mode. The cracking load was 40 kN, which was equivalent to the new specimen NEW2 with a core hole diameter of 160 mm with diagonal bracing, and smaller than that of the traditional specimen EW1. When loaded to 70 kN to draw wall cracks, a “rustling” sound could be heard on the wall that was greater than that of the EW1 specimen. The test phenomenon indicated that compared with the EW1 traditional specimen, the increase in the core hole diameter could delay the damage to the composite wall. The crack development law of specimen EW2 was similar to that of specimen EW1. When the top lateral displacement *δ* reached 9.52 mm (*θ* = 0.680%), an “X”-shaped crack had formed. Compared with the EW1 specimen, the increase in the diameter of the core hole delayed the damage of the specimen. When the top lateral displacement *δ* reached 11.04 mm (*θ* = 0.789%), several diagonal cracks in the middle of the specimen suddenly appeared, and a small piece of the EPSC at the root was broken due to squeezing. At the same time, the maximum crack width reached approximately 6 mm. Then, the loading stopped for safety. The final failure mode of specimen EW2 is shown in Figure 7d. The comparison of crack width at peak load between the four walls is shown in Table 3.

### 3.2. Hysteretic Response

**Figure 8:**
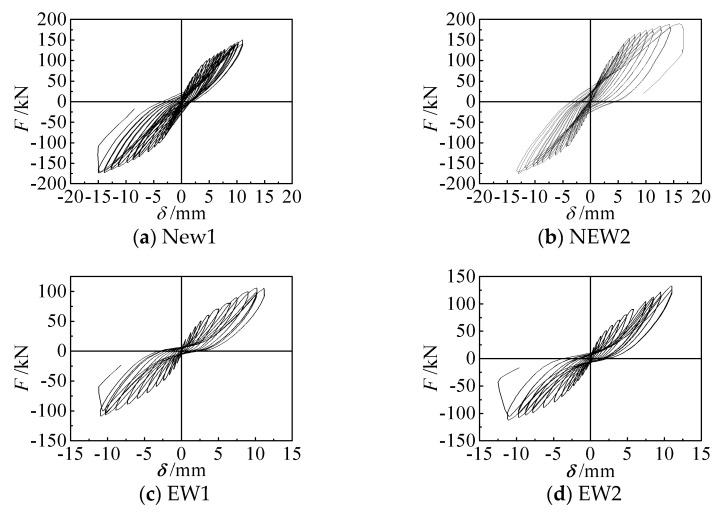
Hysteresis curves of specimens.

The load-displacement (*F-δ*) hysteresis curves at the loading point of each specimen are shown Figure 8a–d. All of the specimens were basically in an elastic working state at the initial stage of loading, and the area of the hysteresis loops was small. As the loading increased, cracks appeared and then gradually extended and expanded, the steel bar yielded, and the areas of the hysteretic loops of each specimen gradually increased. As the lateral displacement increased, and the EPSC and concrete damage intensified, “X”-shaped cracks formed, the stiffness of the specimen degraded significantly, and the residual deformation increased during unloading. At the later stage of loading, there were “pinches” in the hysteresis curves that were worse for the traditional specimens than for the new specimens with diagonal bracing. This indicated that diagonal bracing improved the seismic performance of the EPSC latticed concrete wall when the amount of concrete and steel bars was equal, but when the diameter of the core hole increased, the degrees of improvement of the wall bearing capacity and the elastoplastic deformation capacity were different for walls of different lattice types.

### 3.3. Skeleton Curves

**Figure 9:**
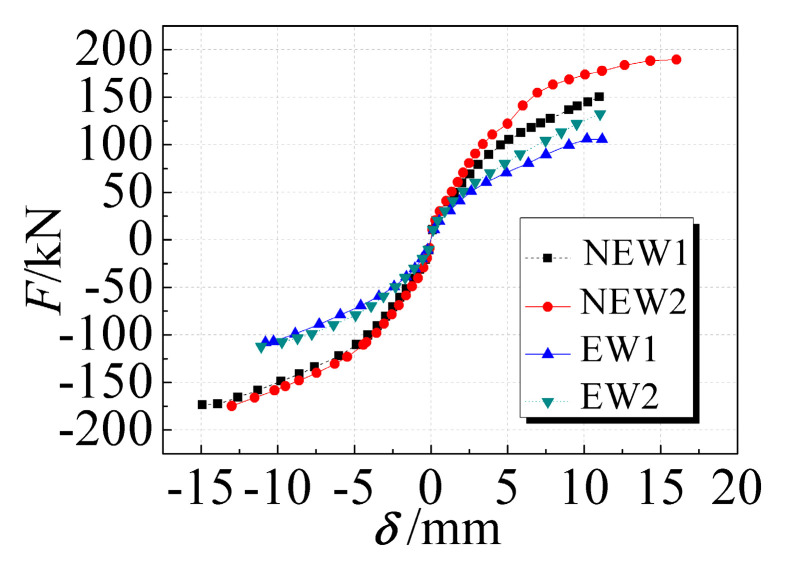
Skeleton curves of specimens.

The comparison of the envelope curves of the four specimens is shown in Figure 9. The skeleton curves of each specimen were relatively close at the initial stage of loading. With the increase in the lateral load, the EPSC and the concrete of the specimens cracked, and the steel bar yielded. The difference between the skeleton curves gradually increased, and the relative positions became clear. For the same lateral displacement, the skeleton curve of the new type of EPSC latticed concrete wall with diagonal bracing was farther away from the X-axis than the traditional types were. The skeleton curve of the specimen with a larger core hole diameter was farther away from the X-axis than the smaller core hole diameter specimens. This indicated that adding diagonal bracing and increasing the diameter of the core hole could improve the seismic performance of the latticed concrete wall.

The cracking, yielding, and failure points of the skeleton curve of each specimen are shown in Table 4. The energy equivalence method [37] was used to calculate the yield load *F*_y_. Taking specimen EW1 and specimen EW2 as examples, the relative value was the ratio of the corresponding values for specimen EW2 and specimen EW1.

The cracking load of each specimen was between 30 kN and 50 kN. Compared with specimens EW1 and EW2, the yield loads of specimens NEW1 and NEW2 were increased by 50% and 48%, and the failure loads were increased by 51% and 49%, respectively. With the addition of diagonal bracing, the yield load and the failure load were improved to varying degrees, indicating that the new type of EPSC latticed concrete wall with diagonal bracing had an improved seismic performance compared with the traditional types when the amount of concrete and steel was equivalent. Compared with specimens NEW1 and EW1, the yield loads of specimens NEW2 and EW2 were increased by 11% and 13%, and the failure loads were increased by 12% and 14%, respectively. This indicates that increasing the diameter of the core hole could slightly increase the yield and failure loads of the latticed concrete wall.

### 3.4. Deformability

The yield displacement *δ*_y_, elastoplastic maximum displacement *δ*_u_, and displacement ductility coefficient μ of each specimen are shown in Table 5. Among them, *δ*_y_ and *δ*_u_ are the mean values of the positive and negative values, *μ* = *δ*_u_/*δ*_y_.

Compared with specimens EW1 and EW2, specimens NEW1 and EW2 had a slight increase in the yield displacement, and the maximum elastoplastic displacement and the displacement ductility coefficient were increased by 17% and 31% as well as 15% and 28%, respectively, indicating that the diagonal bracing could improve the elastoplastic maximum displacement and displacement ductility coefficient of the latticed concrete composite wall effectively, and the improvement was more obvious for the specimens with large core hole diameters.

As the core hole diameter increased, the deformation capacity of the new-type EPSC latticed concrete wall with diagonal bracing was slightly improved compared with that of the traditional concrete walls, and the elastoplastic maximum displacement and the displacement ductility coefficients were increased by 12% and 11%, respectively. This might have been caused by the joint effect of the reinforcement and the lattice type. The reinforcement of specimen EW2 was exactly the same as that of specimen EW1. The reinforcement ratio of specimen EW2 was halved compared with specimen EW1. In addition, the traditional latticed concrete wall could not fully exert the compressive performance of concrete. When the core hole diameter increased, it might have led to the phenomenon of the deformation performance of the composite wall not being improved.

### 3.5. Stiffness Degradation

**Figure 10:**
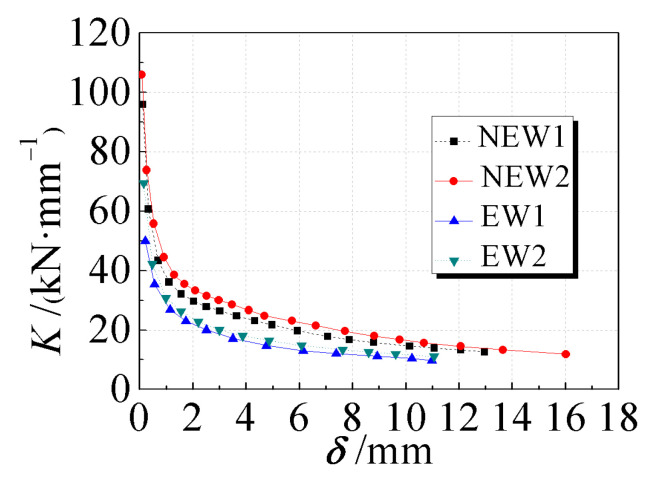
Stiffness degradation curves of specimens.

The average secant stiffness-displacement (*K-δ*) curve of each specimen is shown in Figure 10. These curves represent the stiffness degradation law of each specimen. Compared with specimens EW1 and EW2, the initial stiffness of the specimens NEW1 and NEW2 was increased by 91% and 52%, respectively, indicating that diagonal bracing could significantly increase the initial stiffness of the composite wall. The initial stiffness of the specimens NEW2 and EW2 was increased by 10% and 39%, respectively, compared with the stiffness of the specimens NEW1 and EW1, indicating that the increase in the core hole diameter could improve the initial stiffness of the specimens, and the increase for the traditional type was relatively obvious.

At the later stage of loading, the average secant stiffness tended to be stable. The initial stiffness of the composite wall was mainly contributed by the reinforced concrete skeleton. As the core hole diameter increased, the initial stiffness improved. The traditional latticed concrete wall was improved to a new type of latticed wall with diagonal bracing, which could give full play to the compressive performance of the concrete, and significantly improve the initial stiffness.

### 3.6. Energy Dissipation Capacity

**Figure 11:**
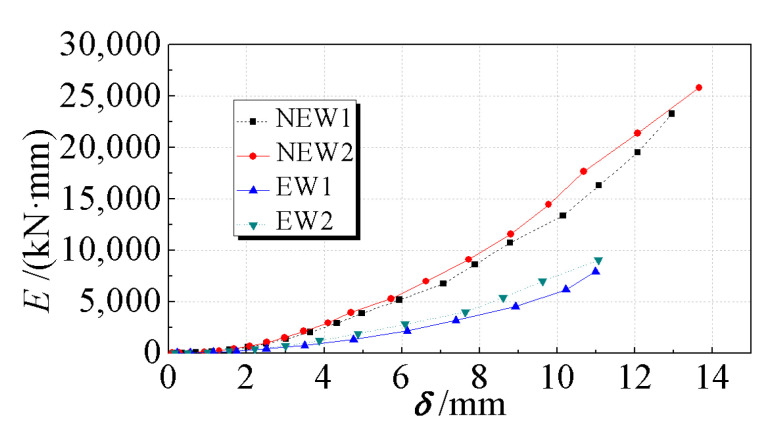
Accumulated energy dissipations of specimens.

The accumulative energy consumption of each specimen was adopted as the standard to compare and evaluate the energy consumption capacities. For the hysteresis curve of each specimen, the cumulative energy consumption during the first cycle under each level of load was used for comparative analysis. A specimen with a fuller hysteresis curve had higher accumulated hysteretic energy consumption and better energy consumption capacity. The cumulative energy dissipation-displacement (*E-δ*) curves of each specimen are shown in Figure 11. The cumulative energy dissipation values of each specimen under the first-level load before specimen failure are listed in Table 6.

The energy dissipation capacity of the new type of EPSC latticed concrete wall with diagonal bracing was better than that of the traditional type for the same displacement, as shown in Figure 11. Compared with specimens EW1 and EW2, the cumulative energy consumption of specimens NEW1 and NEW2 increased by 193% and 186%, respectively, indicating that the energy consumption capacity of the new composite wall with diagonal bracing was significantly increased compared with the traditional composite wall. The core hole diameter increased, and the cumulative energy consumption increased by 11% and 14% for the new and traditional walls, which was not as obvious as the increase in cumulative energy consumption for the optimization of the wall design.

### 3.7. Reinforcement Strain

**Figure 12:**
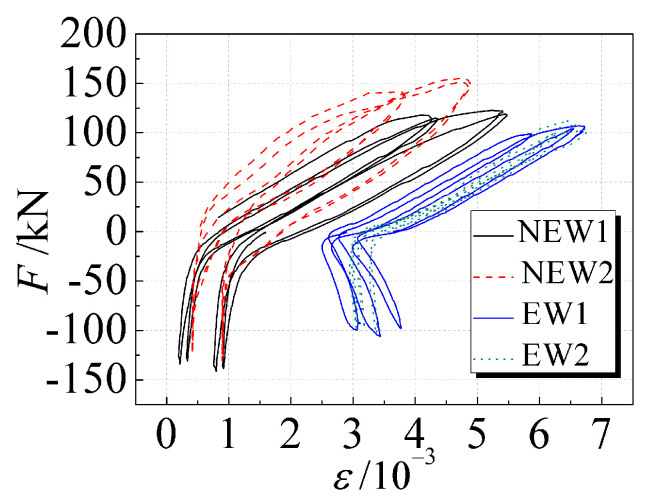
Reinforcement load-strain hysteresis curves at the bottom of the lattice side column.

In the later stage of loading, when “X”-shaped cracks appeared on the wall, the strain measurement point S1 of the vertical reinforcement at the bottom of the lattice side column was selected for analysis. The 13th and 14th cycles were selected for the new specimens with diagonal bracing, and the 10th and 11th cycles were selected for the traditional specimens. Figure 12 shows the corresponding load-strain (*F-ε*) hysteresis curve of each strain measurement point.

At the later stage of loading, the vertical reinforcement bars at the bottom of the lattice side columns of each specimen had all yielded, and the strains were close to or greater than 5000 *με*. The strain of the steel bars of the traditional specimens was generally larger than that of the new specimens with diagonal bracing, indicating that the addition of diagonal bracing delayed the development of the lateral displacement of the wall and improved the seismic performance of the composite wall. The increase in the core hole diameter also slowed down the strain development of the vertical reinforcement at the bottom of the lattice side column.

## 4. Numerical Simulations

### 4.1. Numerical Model

**Figure 13:**
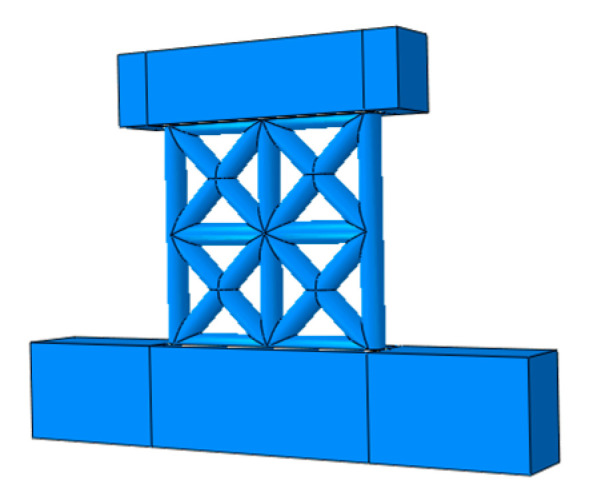
Finite element model of latticed concrete wall specimen with diagonal bracing.

**Figure 14:**
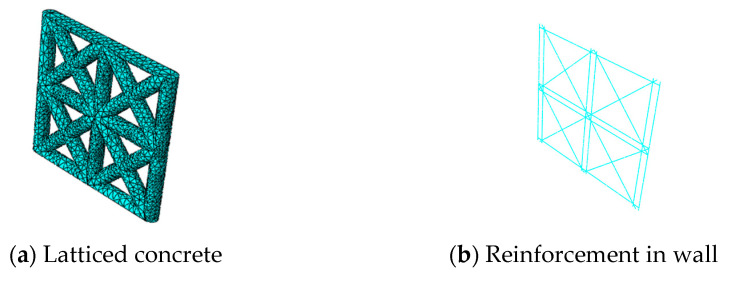
Mesh generation of latticed concrete wall specimen with diagonal bracing.

In order to understand the stress condition of the specimen in the whole process of loading, the finite element software ABAQUS 6.14 [38] was applied and a simplified modeling method was adopted. Ignoring the contribution of EPSC to the seismic performance of the composite wall, a numerical simulation analysis of a pseudo-static test was carried out on a new latticed concrete wall with diagonal bracing whose core hole diameters were 120 mm and 160 mm. The solid element was used to model concrete, and the T3D2 truss element was embedded into the concrete to model reinforcement. The finite element model and the mesh generation of the new latticed concrete wall with diagonal bracing are shown in Figure 13 and Figure 14.

A two-fold line constitutive model [39] was adopted to simulate the reinforcement. The Concrete Damaged Plasticity (CDP) constitutive model that is widely used in ABAQUS was adopted to simulate the concrete. The CDP model is based on the concept of fracture energy and stiffness degradation in continuum damage mechanics, and it introduces two variables of tension and compression damage to establish a plastic damage model of concrete under lateral cyclic loading to describe the different states of concrete damage [40,41]. The concrete and steel parameters of the model are shown in Table 7.

### 4.2. Simulation Results and Comparative Analysis

#### 4.2.1. Skeleton Curve

**Figure 15:**
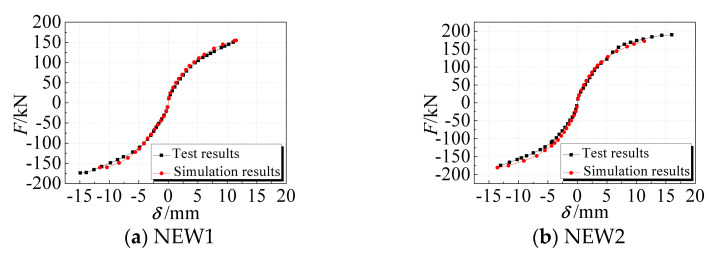
Comparison of test and calculated skeleton curves.

The comparison between the numerical simulation results of the skeleton curve and the test results is shown in Figure 15. The bearing capacity and the deformation capacity of the specimen were mainly analyzed. The comparisons of the test values (mean value of positive and negative) and the numerical simulation values (mean value of positive and negative) of the two specimens are shown in Table 8.

The numerical simulation results were in good agreement with those of the test. The bearing capacity of the numerical simulation was lower than the test value and the difference between them did not exceed 10%. The comparison results indicate that the simplified finite element model is suitable to simulate and analyze the seismic performance of the EPSC latticed concrete wall, even if the contribution of the EPSC to the seismic performance of the composite wall and the damage accumulation during the test loading process are not considered in the model.

#### 4.2.2. Mises Stress

**Figure 16:**
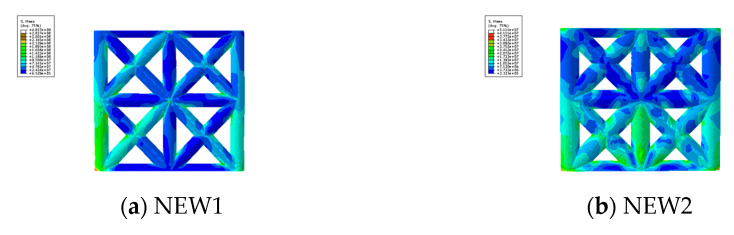
Mises stress of the wall.

The Mises stress cloud images of the specimens NEW1 and NEW2 are shown in Figure 16. The figure indicates that the damage morphology of the two specimens was consistent to a certain extent, and the middle and lower parts of the specimen, especially the side columns and the 45° oblique members, were seriously damaged.

## 5. Conclusions

In this research, four EPSC latticed concrete walls with different lattice types and core hole diameters were investigated with pseudo-static tests and numerical simulations. The following conclusions were obtained based on the analysis of the experimental phenomena, results, and numerical simulation:

(1) In the traditional EPSC latticed concrete wall, the horizontal lattice beam and the vertical lattice column were coordinated to bear the force. After adding diagonal bracing, the wall became a new type of EPSC latticed concrete wall with diagonal bracing. The horizontal lattice beams, vertical lattice columns, and diagonal ribs were coordinated to bear the force, and the compression resistance of the concrete could be fully utilized in the new type wall with diagonal bracing.
(2) The four specimens (traditional type and new type with diagonal bracing) all showed shear failure mode. For the traditional specimens, the wall cracks were relatively short and dense, especially for specimen EW1, and the distribution of “X”-shaped cracks was extremely regular. With the addition of diagonal bracing, the cracking and damage process of the composite wall was slowed down.
(3) Compared with the traditional specimens, the new specimens with diagonal bracing had different degrees of improvement of the bearing capacity, elastoplastic deformation capacity, initial stiffness, and energy dissipation capacity. Initial stiffness and cumulative energy consumption were improved more than 52% and 180%, respectively. In the case of the nearly same amount of concrete, the bearing capacity of the new specimens increased by more than 12%. This indicated that the addition of diagonal bracing could improve the seismic performance of the latticed concrete composite wall.
(4) Different lattice types and core hole diameters had different effects on the seismic performance of the EPSC latticed concrete walls. As the core hole diameter increased 20 mm, the bearing capacity and initial stiffness were improved more than 12% and 10%, respectively. For the same lattice type, the reinforcement was the same, and the increase in the core hole diameter led to a decrease in the reinforcement ratio of the specimen with a larger core hole diameter. The combined effect of the lattice type and the reinforcement ratio might have led to different degrees of improvement in the seismic performance of the composite wall. The study of the change in the core hole diameter could provide a basis for the definition of the use range of walls with different thicknesses in actual projects.
(5) The addition of diagonal bracing could delay the strain development process of the vertical reinforcement at the bottom of the lattice side columns. The numerical simulation results obtained with the simplified modeling method were in good agreement with the experimental results, which indicated that the simplified modeling method is suitable for analyzing the seismic performance of the EPSC latticed concrete wall, and provides useful insights for subsequent parametric research on latticed concrete walls.

EPSC latticed concrete walls could also be used as partition walls, but compared with lightweight steel drywall partitions [42], the wall was heavier. Energy dissipation devices can be adopted to improve the seismic performance of EPSC latticed concrete walls, just as butterfly-shaped (BS) links improving the seismic performance of traditional steel plate shear walls [43]. In the future, studies on the seismic performance of EPSC latticed concrete wall could be carried out by reducing the weight and adding energy-consuming devices.

## Figures and Tables

**Table 1:** Properties of EPSC.

Apparent Density/(kg/m^3^)	Thermal Conductivity /(W·m^−1^·K^−1^)	Compressive Strength/MPa	Splitting Tensile Strength/MPa	Fire Resistance/h
≤380	≤0.08	≥0.40	≥0.10	≥3

**Table 2:** The main parameters of the specimens.

Specimen	Dimension (Height × Width × Thickness)/mm	Core Hole Diameter/mm	Reinforcement		Reinforcement Ratio (%)		Cube Compressive Strength of Concrete *f*_cu_/MPa	Latticed Form
			Latticed Beams, Columns	Diagonal Bracing	Latticed Beams, Columns	Diagonal Bracing		
NEW1	1200 × 1500 × 210	120	2B8	1B8	0.890	0.445	20.8	New type with diagonal bracing
NEW2	1200 × 1500 × 250	160			0.500	0.250		
EW1	1200 × 1500 × 210	120		—	0.890	—		Traditional type
EW2	1200 × 1500 × 250	160			0.500			

**Table 3:** Comparison of crack width at peak load.

Specimen	Crack Width at Peak Load/mm
NEW1	5
NEW2	6
EW1	5
EW2	6

**Table 4:** Test results of characteristic points.

Specimen	Cracking Point	Yield Point			Yield Point		
	*F_cr_*/kN	*F_y_*/kN	Average Value of *F_y_*/kN	Relative Value of *F_y_*	*F_u_*/kN	Average Value of *F_u_*/kN	Relative Value of *F_u_*
NEW1	+49.34	+100.90	108.05	1.000	+150.33	162.00	1.000
		−115.20			−173.66		
NEW2	+40.89	+128.98	120.74	1.117	+189.58	182.21	1.125
		−112.50			−174.84		
EW1	+30.70	+71.17	71.59	1.000	+106.11	107.06	1.000
		−72.01			−108.00		
EW2	+40.85	+84.50	81.25	1.135	+132.46	122.38	1.143
		−78.00			−112.30		

Notes: *F_cr_* = Cracking load; *F_y_* = Yield load; *F_u_* = Failure load.

**Table 5:** Displacement and displacement ductility ratio.

Specimen	*δ_y_*/mm	Relative Value of *δ_y_*	*δ_u_*/mm	Relative Value of *δ_u_*	*μ*	Relative Value of *μ*
NEW1	5.08	1.000	12.96	1.000	2.55	1.000
NEW2	5.12	1.008	14.51	1.120	2.83	1.110
EW1	4.99	1.000	10.99	1.000	2.20	1.000
EW2	5.01	1.004	11.06	1.006	2.21	1.005

Notes: *δ*_y_ = Yield displacement; *δ*_u_ = Failure displacement; μ = *δ*_u_/*δ*_y_.

**Table 6:** Comparison of cumulative energy dissipation.

Specimen	Cumulative Energy Dissipation *E*/(kN·mm)	Relative Value of Cumulative Energy Dissipation
NEW1	23,282	1.000
NEW2	25,848	1.110
EW1	7923	1.000
EW2	9030	1.140

**Table 7:** Material parameters.

Material Parameters	Wall Concrete	Wall Reinforcement	Material Parameters	Wall Concrete
Elastic modulus E (GPa)	26.00	200	Initial yield compressive stress σ_co_ (MPa)	8.60
Poisson’s ratio υ	0.20	0.3	Compression variable ω_c_	1.00
Density ρ (kg/m^3^)	2500	7800	Stretch variable ω_t_	0.00
Divergence angle ψ (°)	30		Damping ratio ξ	0.05

**Table 8:** Comparisons between test results and numerical simulation results.

Specimen	*V*_E_ (kN)	*V*_N_ (kN)	*V*_E_/*V*_N_
NEW1	162.00	157.50	1.02
NEW2	182.21	176.45	1.03

Notes: *V*_E_ = Test results; *V*_N_ = Numerical simulation results.

## Data Availability

Not applicable.
